# Author Correction: Targeted inhibition of ubiquitin signaling reverses metabolic reprogramming and suppresses glioblastoma growth

**DOI:** 10.1038/s42003-024-07075-8

**Published:** 2024-10-29

**Authors:** Rossella Delle Donne, Rosa Iannucci, Laura Rinaldi, Luca Roberto, Maria A. Oliva, Emanuela Senatore, Domenica Borzacchiello, Luca Lignitto, Giorgio Giurato, Francesca Rizzo, Assunta Sellitto, Francesco Chiuso, Salvatore Castaldo, Giovanni Scala, Virginia Campani, Valeria Nele, Giuseppe De Rosa, Chiara D’Ambrosio, Corrado Garbi, Andrea Scaloni, Alessandro Weisz, Concetta Ambrosino, Antonella Arcella, Antonio Feliciello

**Affiliations:** 1 Department of Molecular Medicine and Medical Biotechnology, University Federico II, Naples, Italy; 2 Biogem, Ariano Irpino, Avellino, Italy; 3 I.R.C.C.S Neuromed, Pozzilli (Isernia), Italy; 4 Laboratory of Molecular Medicine and Genomics, Department of Medicine, Surgery and Dentistry SMS, University of Salerno, Salerno, Italy; 5 Department of Biology, University Federico II, Naples, Italy; 6 Department of Pharmacy, University Federico II, Naples, Italy; 7 Proteomics, Metabolomics and Mass Spectrometry Laboratory, ISPAAM, National Research Council, Portici (Naples), Italy; 8 Genome Research Center for Health, Campus of Medicine, University of Salerno, Salerno, Italy; 9 Department of Science and Technology University of Sannio, Benevento, Italy

**Keywords:** Cancer metabolism, Kinases

Correction to: *Communications Biology* 10.1038/s42003-022-03639-8, published online 02 August 2022

The original version of this Article contained a repetition of two images in Fig. 5f, in which the FACS plots for SANPs-siRNAc and SANPs-siPraja2 were identical. The correct version of Fig. 5 is:
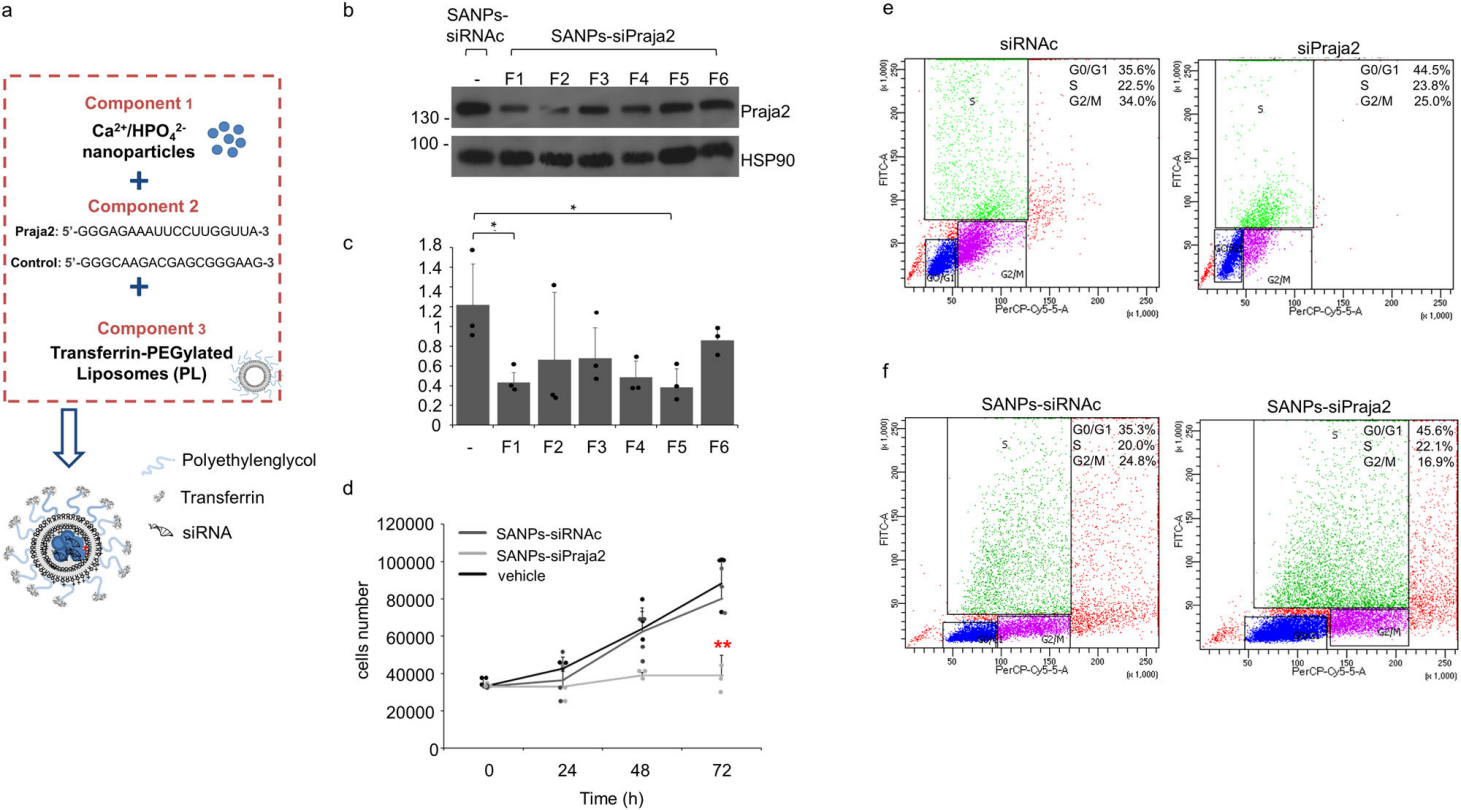


This has now been corrected in both the PDF and HTML versions of the Article.

