# Supplementary material for: Targeted inhibition of ubiquitin signaling reverses metabolic reprogramming and suppresses glioblastoma growth
Source: Commun Biol. 2022 Aug 2;5:780. doi: 10.1038/s42003-022-03639-8 (PMC9345969; doi:10.1038/s42003-022-03639-8)
Supplement: Supplementary file 2 — Supplementary Information [file 42003_2022_3639_MOESM2_ESM.pdf]

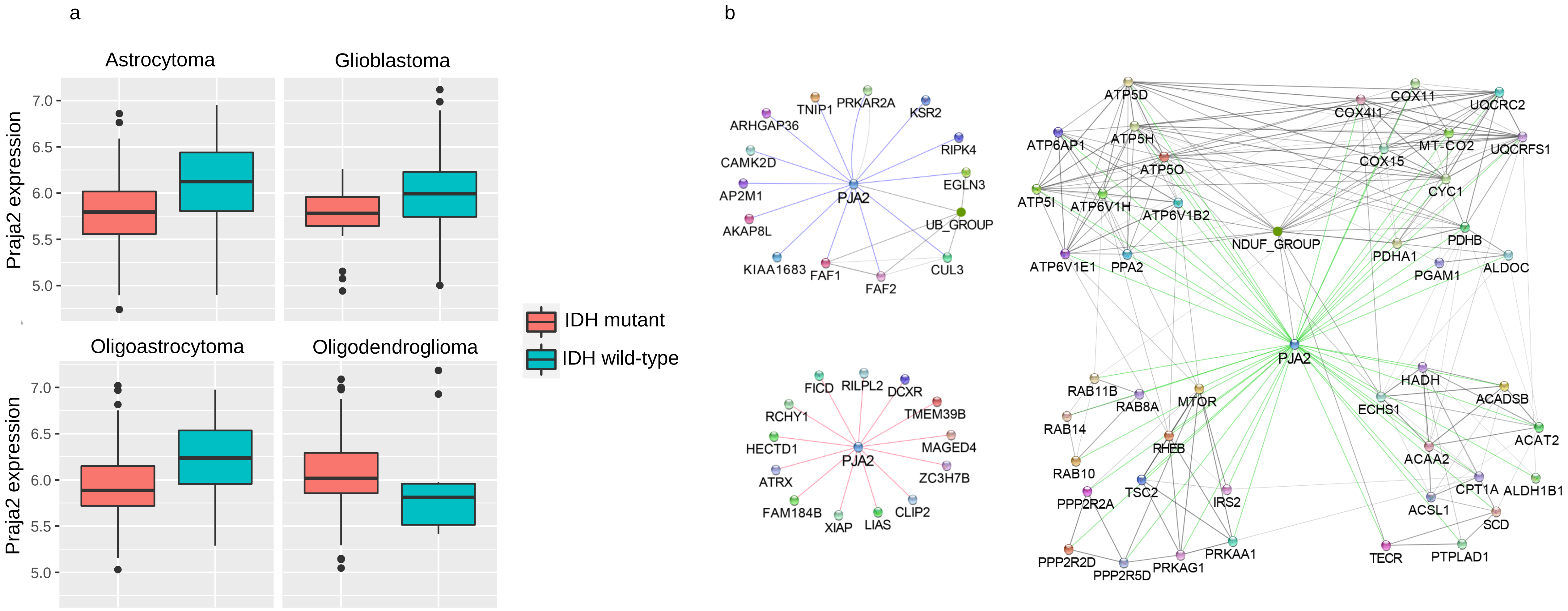

Supplementary Fig1: a Boxplots comparing Praja2 expression between IDH wild-type and mutant in different histological subtypes of glioma samples from the TCGA-GBM cohort. **b** Separate Praja2 protein-protein interaction (PPI) network. Intact interactors (blue edges, upper left panel), interactors identified in this study (green edges) and STRING interactors (grey edges, right panel), GBM-ARACNE interactors (red edges,lower panel)

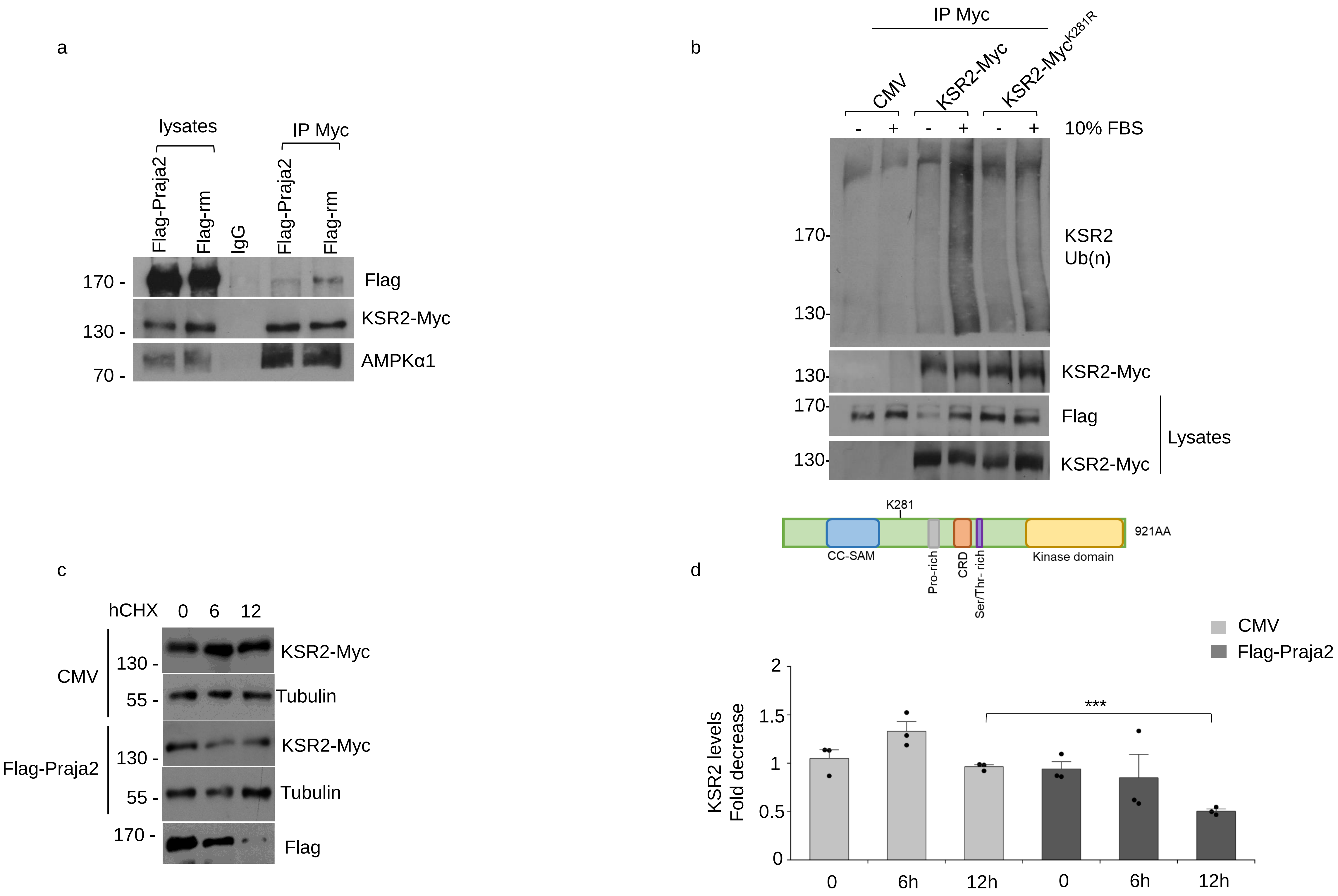

Supplementary Fig2: a. A trimeric complex composed of praja2, KSR2 and AMPKα1 was isolated from lysates of U87MG cells transiently expressing Flag-praja2 and KSR2-Myc and subjected to immunoprecipitation with anti-Myc antibody. Lysates were immunoblotted with the indicated antibodies. **b. Upper panel** HEK293 cells were transfected with HA–ubiquitin, Flag–praja2 and CMV, KSR2-Myc or $KSR2^{K281R}$. After 24 hours cells were serum deprived for 18 hours and then treated with 10 μM MG132 and serum stimulated (FBS 10%) for 5 hours. Lysates were immunoprecipitated with anti-Myc antibody. Lysates and precipitates were immunoblotted with anti-HA, anti-Flag and anti-Myc antibodies. Representative set of two independent experiments that give the same results is shown. **Lower panel** Schematic rappresentation of KSR2 protein. The ubiquitylated lysine residue was available on *https://www.phosphosite.org* database (Guo A **CST Curation Set:** 3576; **Year:** 2007)**. c.** HEK293 were transiently transfected with CMV and Flag-Praja2 vectors and treated with chycloeximide 10μm at indicate times. Lysates were immunoblotted with the indicated antibodies. **d**. Quantitative analysis of data shown in panel c. A mean value ± S.E.M. of three independent experiments is reported. P-value *** = 0.00016

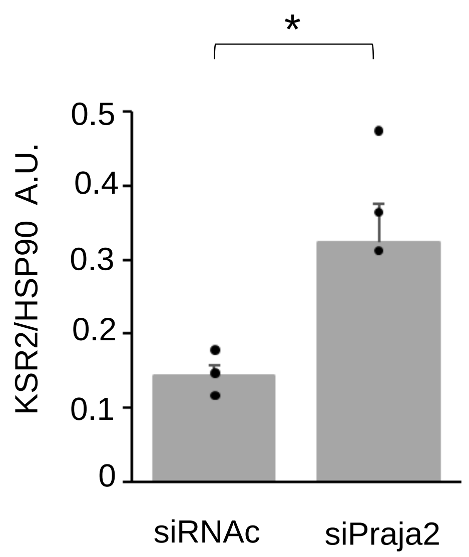

Supplementary Fig3: Quantitative analysis of KSR2 levels of the experiment shown in figure **3E**. A mean value ± S.E.M. of three independent experiments is reported. P-value * = 0.0139

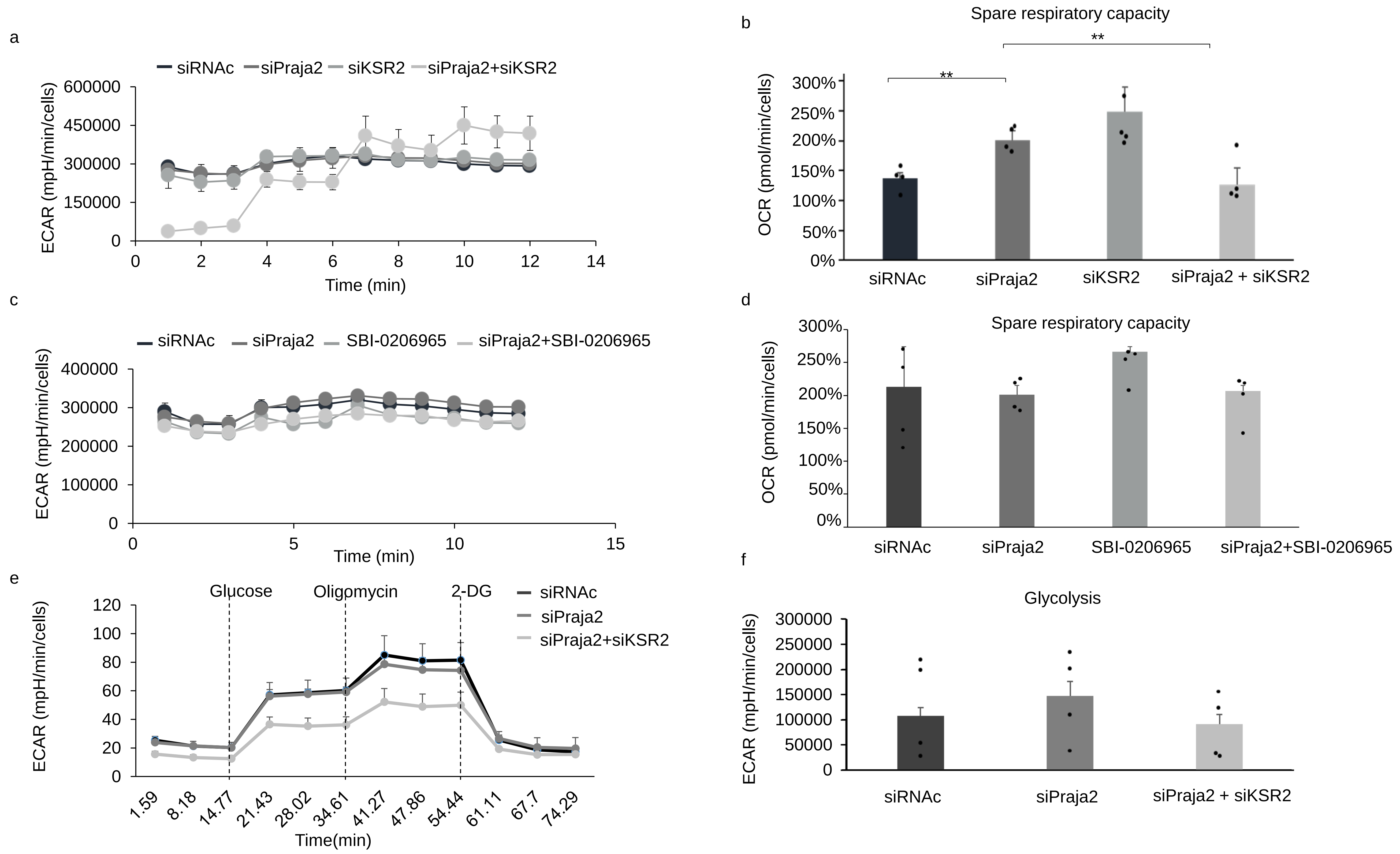

Supplementary Fig4: a. Kinetic profile of extracellular acidification rate (ECAR) in control and silenced cells. Reported data are the mean values ± S.E.M. of four independent experiments. **b.** Spare respiratory capacity was calculated as a percentage value in U87MG silenced cells, as indicated. P value **= 0.0062; **= 0.0019. **c.** Kinetic profile of extracellular acidification rate (ECAR) in control and Praja2-silenced cells. When indicated, cells were treated with the AMPK inhibitor SBI-0206965 (5 μM for 4 h). **d.** Spare respiratory capacity was calculated as a percentage value in control and Praja2-silenced cells. When indicated, cells were treated with the AMPK inhibitor SBI-0206965 (5 μM for 4 h). **e.** Kinetic profile of extracellular acidification rate (ECAR) in control and silenced cells. Reported data are the mean values ± S.E.M. of four independent experiments. **f.** Indices of glycolysis function were calculated from ECAR profile: Glycolytic rate corresponds to maximal rate measurement before oligomycin injection, minus last rate measurement before glucose injection.

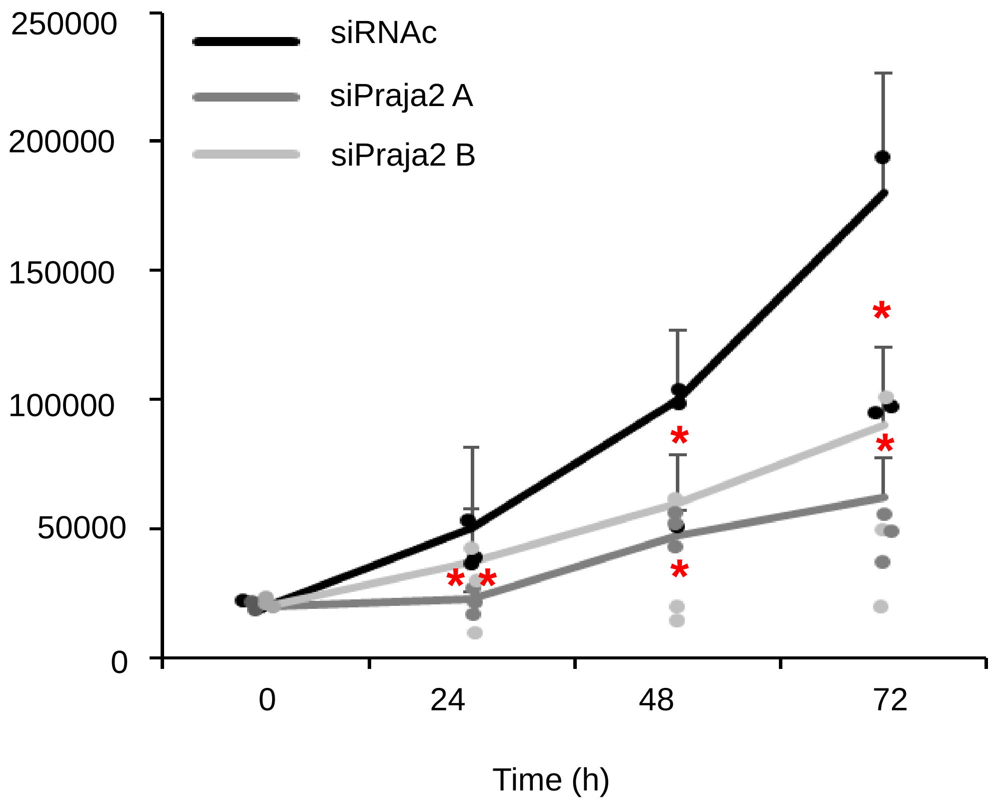

Supplementary Fig5: Growth curves of U87MG cells treated with control or two independent pair of Praja2 si-RNAs. At indicated time points, cells were harvested and counted. Three independent experiments were performed and the corresponding mean values ± S.E.M are shown. P-value: ** = 0.0019; * = 0.025; * = 0.018; * = 0.015; * = 0.029.

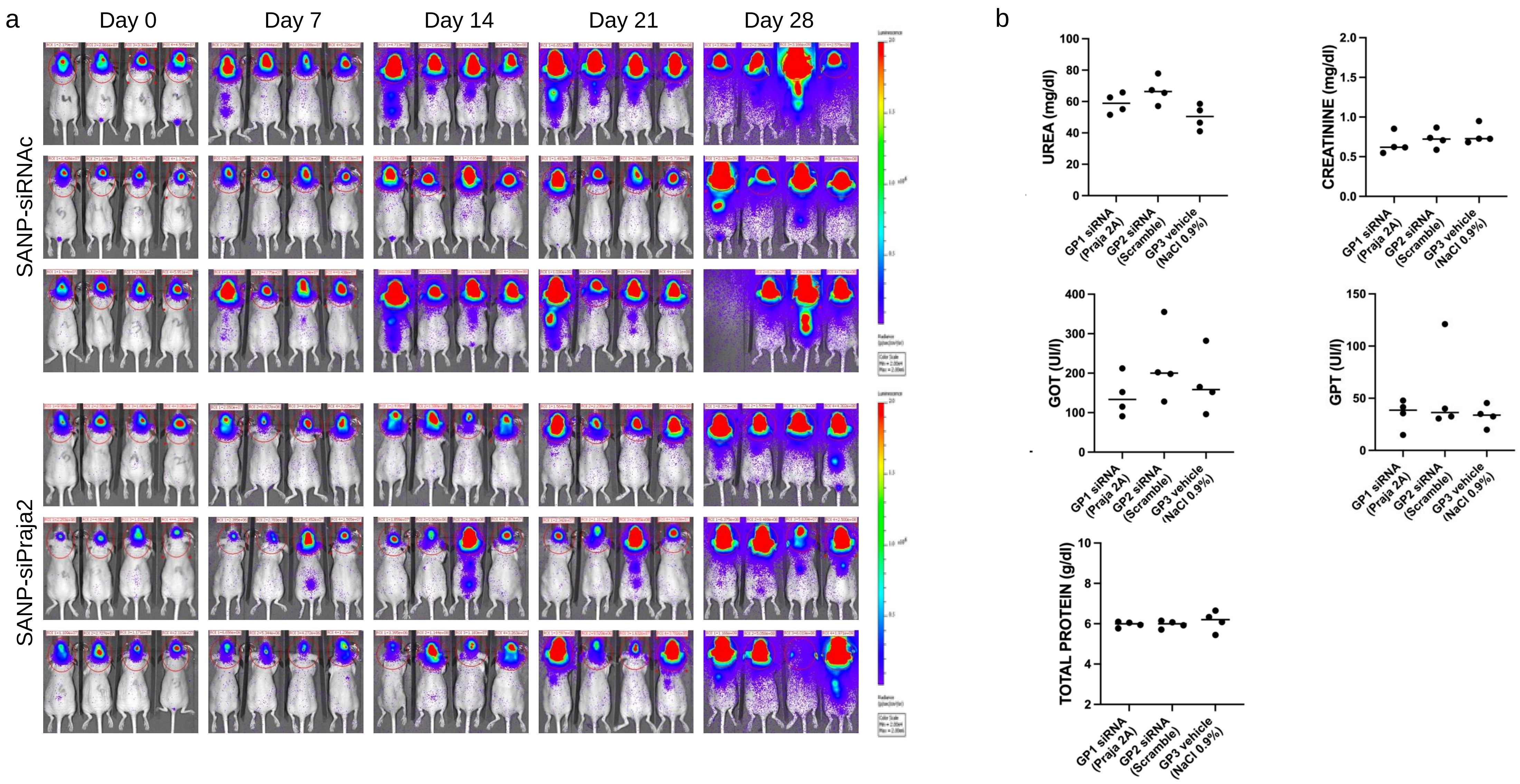

Supplementary Fig 6: a.U87MG-Luc cells were injected into the brain of 6 weeks-old CD1 mice. Three hours following implantation, bioluminescent intensity (BLI) was measured by intraperitoneal injection of 150 mg/kg D-Luciferin potassium salt. At 1-week post-injection, based on BLI measurement, mice were randomized into 2 experimental groups of 12 animals, and each group was treated by tail vein injection with SANPs-siPraja2 (GP1) and SANPs-siRNAc (GP2), respectively. Treatments were repeated twice a week for 4 weeks, then 4 mice for each group were sacrificed and organs collected. BLI analysis was performed every week and quantitative data collected. **b.** Organ functions were monitored by biochemical tests on plasma samples from nanpoparticles-treated mice.

Upstream Regulator	Molecule Type	Predicted Activation State	Activation z-score	Target Molecules in Dataset
DDX5	enzyme	Activated	4,899	ATP5MC1,ATP5MC3,ATP5ME,ATP5MG,ATP5PB,ATP5PD,ATP5PF,COX6C,COX7A2,COX7B,COX7C,CYCS, NDUFA1,NDUFA4,NDUFA5,NDUFA6,NDUFAB1,NDUFB1,NDUFB2,NDUFB3,NDUFB6,NDUFS5,UQCRB,UQCRQ
CAB39L	kinase	Activated	3,873	ATP5F1E,ATP5MC3,ATP5ME,ATP5MF,ATP5MG,COX17,COX6C,COX7C,NDUFA1,NDUFA4,NDUFB2,NDUFB3, NDUFS5,UQCRB,UQCRQ
IGF1R	transmembrane receptor	Activated	3,162	ATP5MC1,ATP5MC3,ATP5MF,ATP5MG,ATP5PD,ATP5PF,ATP5PO,CYCS,MT-ATP6,NDUFA1,NDUFA4
INSR	kinase	Activated	3,148	ATP5MC1,ATP5MC3,ATP5MF,ATP5MG,ATP5PD,ATP5PF,ATP5PO,CYCS,NDUFA1,NDUFA4
STK11	kinase	Activated	2,646	ATP5MC1,ATP5MC3,ATP5MF,COX7A2,NDUFA4,NDUFA8,UQCRQ
Esrra	transcription regulator	Activated	2,646	ATP5MC1,ATP5MC3,ATP5MF,ATP5PB,ATP5PF,ATP5PO,CYCS,NDUFA5
VEGFA	growth factor	Activated	2,449	ATP5MC3,ATP5MF,ATP5PO,CYB5B,NDUFA1,UQCRB
RB1	transcription regulator	Activated	2,449	COX17,COX7A2,NDUFA4,NDUFA5,NDUFA6,UQCRQ
Hbb-b1	transporter	Activated	2,449	ATP5MC1,ATP5MF,ATP5PO,NDUFA4,NDUFA5,NDUFA6
HBA1/HBA2	transporter	Activated	2,449	ATP5MC1,ATP5MF,ATP5PO,NDUFA4,NDUFA5,NDUFA6
PPARGC1A	transcription regulator	Activated	2,349	ATP5MF,ATP5PD,ATP5PO,COX6C,COX7B,CYCS
FOXO1	transcription regulator	Activated	2	ATP5MC3,ATP5PF,CYCS,NDUFA4,NDUFA8
TFE3	transcription regulator	Activated	2	ATP5PO,CYCS,MT-ATP6,MT-ND5
PPARGC1B	transcription regulator		1,981	ATP5MF,ATP5PO,CYCS,MT-ATP6
PTEN	phosphatase		-0,762	ATP5MC1,ATP5ME,MT-ATP6,NDUFB3
OGT	enzyme		-1,342	COX6C,NDUFA5,NDUFA6,NDUFA8,NDUFB6
TP53	transcription regulator		-1,667	ATP5MC1,ATP5MC3,ATP5PB,COX11,COX7A2,MT-ND5,NDUFA4,NDUFA8,UQCRQ
FLCN	other		-1,998	ATP5PO,CYCS,MT-ATP6,MT-ND5
WNT3A	cytokine	Inhibited	-2,236	ATP5MC3,ATP5PO,NDUFA4,NDUFB2,NDUFB6
CA9	enzyme	Inhibited	-2,236	ATP5ME,ATP5MG,ATP5PD,ATP5PF,ATP5PO
CTNNB1	transcription regulator	Inhibited	-2,236	ATP5MC3,ATP5PO,NDUFA4,NDUFB2,NDUFB6
OGA	enzyme	Inhibited	-2,449	ATP5ME,ATP5MG,COX6C,NDUFA4,NDUFA5,NDUFA6
HIF1A	transcription regulator	Inhibited	-2,449	ATP5MC3,ATP5PF,ATP5PO,NDUFA4,NDUFB2,NDUFB6
KDM5A	transcription regulator	Inhibited	-2,449	COX17,COX7A2,NDUFA4,NDUFA5,NDUFA6,UQCRQ
NR4A1	ligand-dependent nuclear receptor	Inhibited	-2,828	ATP5MC3,ATP5PF,COX11,COX7A2,NDUFA1,NDUFA6,NDUFB3,UQCRQ
BACH1	transcription regulator	Inhibited	-2,828	ATP5MC1,ATP5MC3,ATP5PF,ATP5PO,NDUFA6,NDUFAB1,NDUFB6,UQCRB
CPT1B	enzyme	Inhibited	-3,961	ATP5ME,ATP5MG,ATP5PD,COX17,COX7B,NDUFA1,NDUFA4,NDUFA5,NDUFA6,NDUFA8,NDUFAB1,NDUFB2, NDUFB3,NDUFB6,UQCRB,UQCRQ
RICTOR	other	Inhibited	-4,796	ATP5MC1,ATP5MC3,ATP5MF,ATP5MG,ATP5PB,ATP5PD,ATP5PF,ATP5PO,COX11,COX17,COX7A2,COX7B, NDUFA1,NDUFA4,NDUFA5,NDUFA6,NDUFA8,NDUFAB1,NDUFB2,NDUFB3,NDUFB6,UQCRB,UQCRQ

Supplementary Table 1: Upstream regulatorpredicted by Ingenuity Pathway Analysis (IPA). Orange indicates activated upstream regulators (Z-Score>2, pvalue<0.05), while lightblue indicates those that are inhibited (Z-Score<-2, pvalue<0.05)

Sample code	Sample Name	Raw reads
1	u87 siCNT #1	33,898,034
2	u87 siCNT #2	34,372,652
3	u87 siCNT #3	33,853,488
4	u87 siPJA 2 #1	34,497,510
5	u87 siPJA 2 #2	32,930,816
6	u87 siPJA 2 #3	37,208,922

Supplementary Table 2. List of samples processed and raw reads obtained

Formulation	Lipid composition			DSPE-PEG-Mal/CER-p/pPEG	Lipid ratio (mM)	Theoretical loading mg siRNA/mg lipids
SANP 1	DOTAP	CER-PEG	-	-	1:0.125	65
SANP 2	DOTAP	DSPE-PEG	-	-	1:0.125	65
SANP 3	DC-CHOL	DSPE-PEG	-	-	1:0.125	65
SANP 4	DC-CHOL	CER-PEG	-	-	1:0.125	65
SANP 5	DOTAP	DSPE-PEG	CHOL	-	1:0.125:1.8	65
SANP 6	DOTAP	CER-PEG	CHOL	-	1:0.125:1.8	65
SANP 1 (2)	DOTAP	CER-PEG	-	1:50	1:0.125	194
SANP 1(2)-Tf	DOTAP	CER-PEG	-	1:50	1:0.125	194

Supplementary Table 3: Composition of SANPs

	Lipid composition (mM)							
**SANPs**	**DOTAP**	**DcChol**	**CHOL**	**DSPE-PEG**	**CER-PEG**	**diameter (nm ± ds)**	**PI ± ds**	**ζ (mV) ± ds**
SANP 1	1	-	-	-	0.125	120.8 ± 4.0	0.1 ± 0.0	34.7± 5.2
SANP 2	1	-	-	0.125	-	124.7 ± 0.1	0.1 ± 0.0	14.4 ± 3.4
SANP 3	-	1	-	0.125	-	159.5 ± 0.2	0.2 ±0.0	29.8 ± 4.2
SANP 4	-	1	-	-	0.125	132.5 ± 0.3	0.1 ± 0.0	35.6 ±5.8
SANP 5	1	-	1.8	0.125	-	115.0 ± 0.1	0.1 ± 0.0	22.9 ± 4.7
SANP 6	1	-	1.8	-	0.125	118.8 ± 0.4	0.1 ± 0.0	36.8 ± 4.2

Supplementary Table 4: Characterization of blank SANPs: size, polidisperity index (PI) and zeta potential (ζ).

Formulation	diameter (nm ± ds)	PI ± ds	ζ (mV) ± ds	Actualloading (mg siRNA/mg lipids)	EE (%± ds)
SANP 1-**Praja2**	143.5 ± 0.3	0.2 ± 0.1	16.2 ± 1.6	63.5 ± 0.0	97.9 ± 0.0
SANP 2-**Praja2**	114.9 ± 0.1	0.1 ± 0.0	-18.7 ± 2.4	61.3± 2.8	94.4 ± 2.3
SANP 3-**Praja2**	204.4 ±0.3	0.3 ± 0.1	8.2 ± 3.4	64.9± 0.0	100 ± 0.0
SANP 4-**Praja2**	134.6 ± 5.7	0.2 ± 0.1	18.5 ± 6.2	61.3± 2.7	94.4 ± 4.2
SANP 5-**Praja2**	136.7 ± 3.7	0.1 ± 0.1	21.9 ± 3.8	62.0± 1.8	95.6 ± 2.8
SANP 6-**Praja2**	147.1 ± 0.3	0.2 ± 0.1	10.1 ± 0.3	62.8 ± 0.9	96.8 ± 1.4
SANP 1**- (2) Control**	158.6 ± 1.2	0.2 ± 0.1	-13.4 ± 0.7	193.3 ± 3.8	97.8 ± 1.9
SANP 1**- (2) Praja2**	145.9 ± 6.5	0.2 ± 0.01	-18.0 ± 1.9	185.4 ± 2.7	96.2 ± 1.4
SANP 1**- (2) Tf Praja2**	152.7± 4.2	0.3± 0.05	-22.0 ± 2.4	184.8± 0.4	94.7 ± 0.2

Supplementary Table 5: Characterization of SANP-siRNA : size, polidisperity index (PI), zeta potential (ζ), siRNAactual loading and siRNA encapsulation efficiency (EE%)
