# Supplementary material for: Targeted inhibition of ubiquitin signaling reverses metabolic reprogramming and suppresses glioblastoma growth
Source: Commun Biol. 2022 Aug 2;5:780. doi: 10.1038/s42003-022-03639-8 (PMC9345969; doi:10.1038/s42003-022-03639-8)
Supplement: Supplementary file 3 — Description of Additional Supplementary Files [file 42003_2022_3639_MOESM3_ESM.pdf]

Description of Additional Supplementary Files

File name: Supplementary Data 1
Description: Identification details of the proteins identified in this study. Reported are the information on protein false discovery rate (FDR) confidence, protein accession, description, gene name, exp. q-value, sum posterior error probability (PEP) score, sequence coverage (%), number of identified peptides, peptide spectrum matches (PSMs), number of identified unique peptides, number of amino acids, molecular mass, pI, Mascot identification score values, Found in sample, protein groups and modification(s). Specific information on the identified peptides for each protein are also reported, including checked item, identification confidence, annotated sequence, modification, Qvality PEP, Qvality q-value, protein groups, proteins, PSMs, master protein accession, position in master protein, modification in master protein, missed cleavage, theor. MH+, confidence, charge, m/z, delta mass, Percolator q-values, Percolator PEP value, Mascot ion score, sequence in protein and position in protein.

File name: Supplementary Data 2
Description: Raw data of all experiments and uncropped blots.
