# Supplementary material for: Targeted inhibition of ubiquitin signaling reverses metabolic reprogramming and suppresses glioblastoma growth
Source: Commun Biol. 2022 Aug 2;5:780. doi: 10.1038/s42003-022-03639-8 (PMC9345969; doi:10.1038/s42003-022-03639-8)
Supplement: Supplementary file 5 — Supplementary Data 2 [file 42003_2022_3639_MOESM5_ESM.zip › Supplementary Data 2/FIG 5E/siCTRL.pdf]

BD FACSDiva 8.0.1

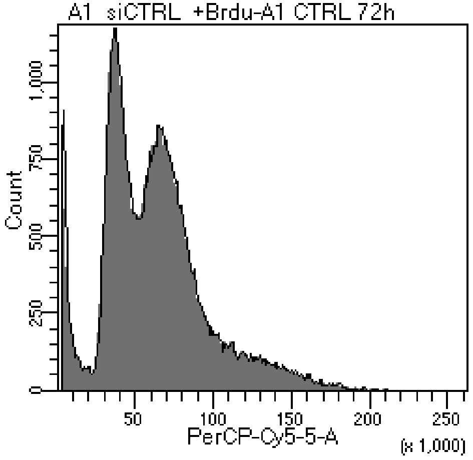

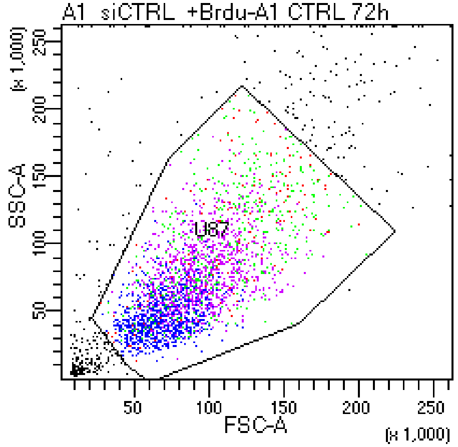

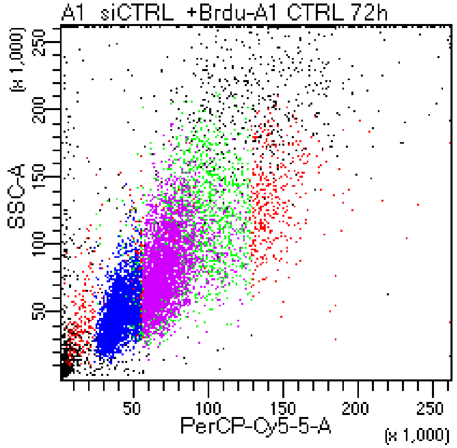

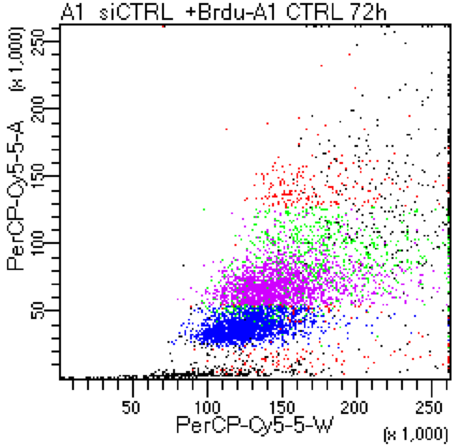

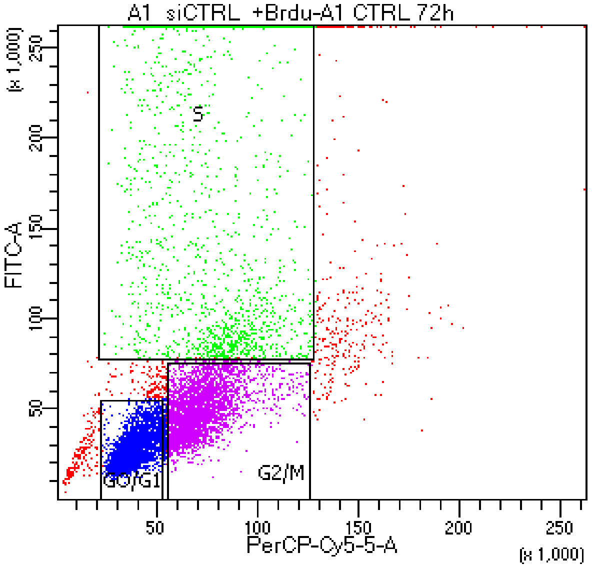

BD FACSDiva 8.0.1

Tube: A1 CTRL 72h

Population	#Events	%Parent	%Total
All Events	59,377	####	100.0
U87	51,212	86.2	86.2
S	11,518	22.5	19.4
GO/G1	18,254	35.6	30.7
G2/M	17,403	34.0	29.3

Experiment Name: Nanoparticelle+/- siPRAJA i...
Specimen Name: A1 siCTRL +Brdu
Tube Name: A1 CTRL 72h
Record Date: Jun 14, 2021 4:16:39 PM
$OP: Administrator
GUID: 737706fe-6eab-4a0c-b59a-...

Population	#Events	%Parent	FITC-A Mean	PerCP-C... Mean
U87	51,212	86.2	77,538	61,383
S	11,518	22.5	193,376	76,309
GO/G1	18,254	35.6	28,288	37,458
G2/M	17,403	34.0	49,211	71,201
