# Supplementary material for: Targeted inhibition of ubiquitin signaling reverses metabolic reprogramming and suppresses glioblastoma growth
Source: Commun Biol. 2022 Aug 2;5:780. doi: 10.1038/s42003-022-03639-8 (PMC9345969; doi:10.1038/s42003-022-03639-8)
Supplement: Supplementary file 5 — Supplementary Data 2 [file 42003_2022_3639_MOESM5_ESM.zip › Supplementary Data 2/FIG 5E/siPraja.pdf]

BD FACSDiva 8.0.1

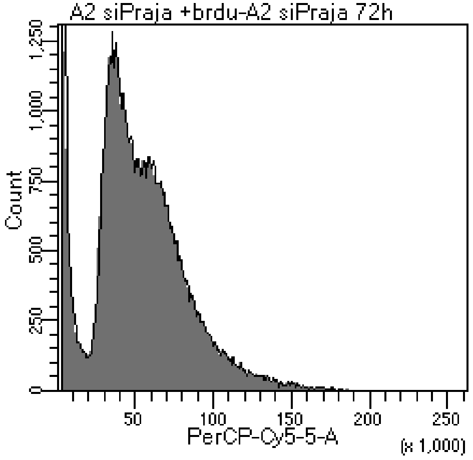

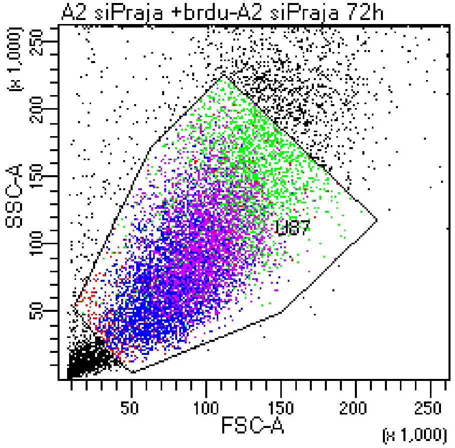

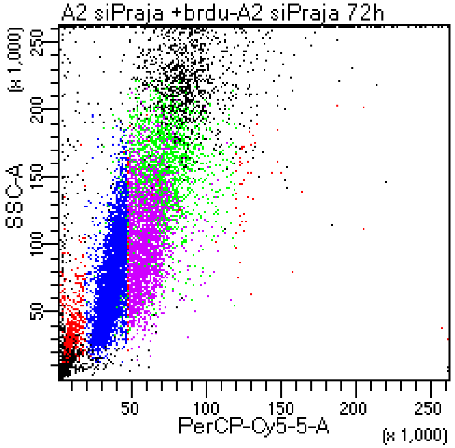

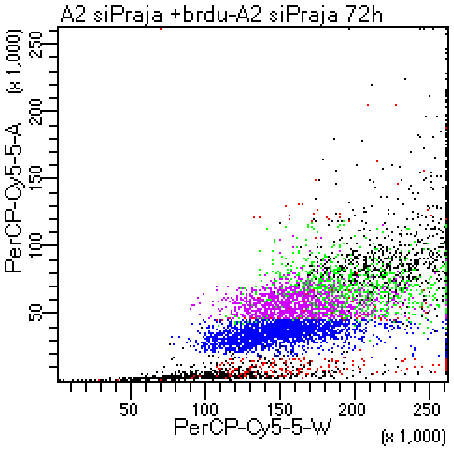

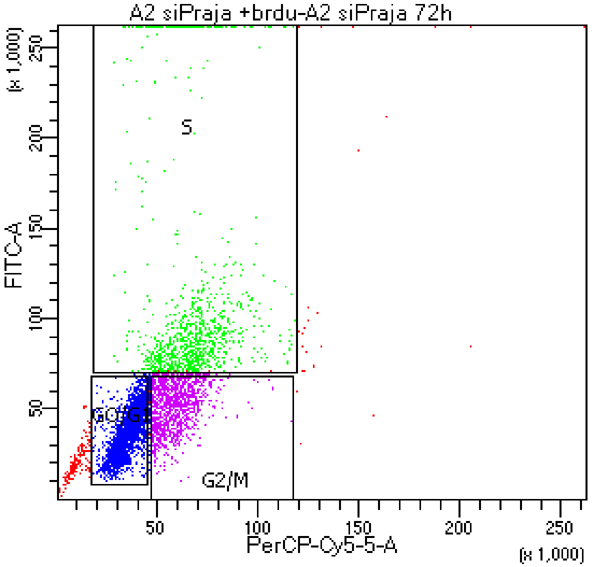

Tube: A2 siPraja 72h

Population	#Events	%Parent	%Total
All Events	64,480	####	100.0
U87	46,459	72.1	72.1
S	11,078	23.8	17.2
GO/G1	20,696	44.5	32.1
G2/M	11,624	25.0	18.0

Experiment Name:	Nanoparticelle+/- siPRAJA i...
Specimen Name:	A2 siPraja +brdu
Tube Name:	A2 siPraja 72h
Record Date:	Jun 14, 2021 4:28:07 PM
$OP:	Administrator
GUID:	2b7c85ef-a8fc-4191-9979-9...

Population	#Events	%Parent	FITC-A Mean	PerCP-C... Mean
U87	46,459	72.1	59,196	47,656
S	11,078	23.8	114,390	66,170
GO/G1	20,696	44.5	36,758	33,943
G2/M	11,624	25.0	51,429	58,408
