# Supplementary material for: Targeted inhibition of ubiquitin signaling reverses metabolic reprogramming and suppresses glioblastoma growth
Source: Commun Biol. 2022 Aug 2;5:780. doi: 10.1038/s42003-022-03639-8 (PMC9345969; doi:10.1038/s42003-022-03639-8)
Supplement: Supplementary file 5 — Supplementary Data 2 [file 42003_2022_3639_MOESM5_ESM.zip › Supplementary Data 2/FIG 5F/SANPs PRAJA2.pdf]

BD FACSDiva 8.0.1

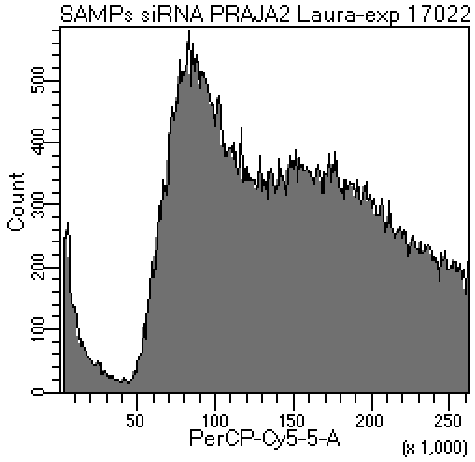

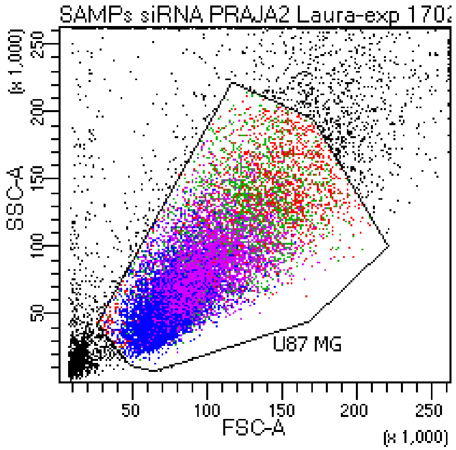

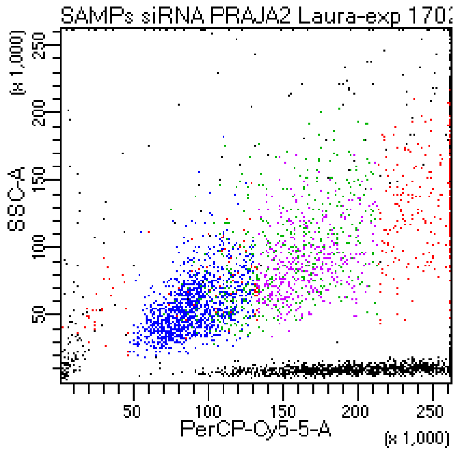

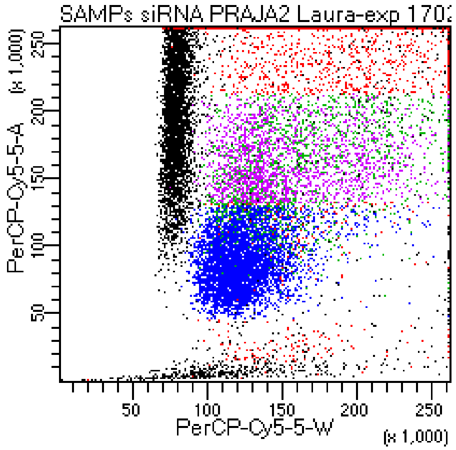

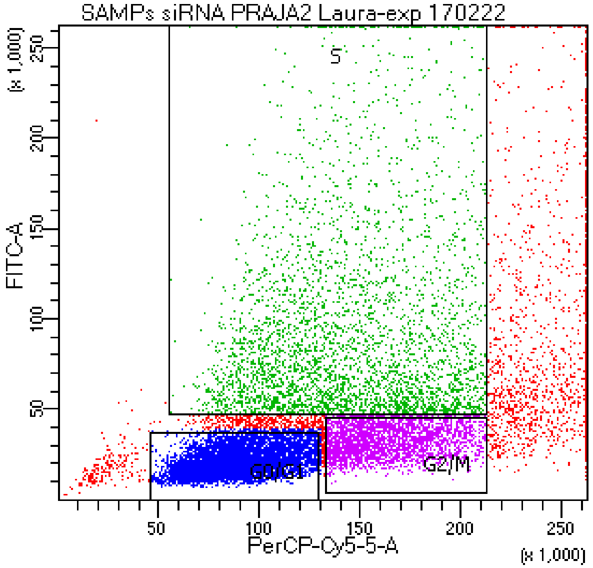

BD FACSDiva 8.0.1

Tube: exp 170222

Population	#Events	%Parent	%Total
All Events	87,361	####	100.0
U87 MG	49,632	56.8	56.8
S	10,965	22.1	12.6
G0/G1	22,621	45.6	25.9
G2/M	8,400	16.9	9.6

Experiment Name:	U87 SAMPs -nanoparticelle...
Specimen Name:	SAMPs siRNA PRAJA2 Laura
Tube Name:	exp 170222
Record Date:	Feb 17, 2022 4:45:12 PM
$OP:	Administrator
GUID:	d471d13a-077d-41f5-95bb-...

Population	#Events	%Parent	FITC-A Mean	PerCP-C... Mean
U87 MG	49,632	56.8	47,334	130,402
S	10,965	22.1	95,253	144,476
G0/G1	22,621	45.6	20,261	87,194
G2/M	8,400	16.9	32,082	163,873
