# Supplementary material for: Targeted inhibition of ubiquitin signaling reverses metabolic reprogramming and suppresses glioblastoma growth
Source: Commun Biol. 2022 Aug 2;5:780. doi: 10.1038/s42003-022-03639-8 (PMC9345969; doi:10.1038/s42003-022-03639-8)
Supplement: Supplementary file 5 — Supplementary Data 2 [file 42003_2022_3639_MOESM5_ESM.zip › Supplementary Data 2/FIG 5F/SANPs scramble.pdf]

BD FACSDiva 8.0.1

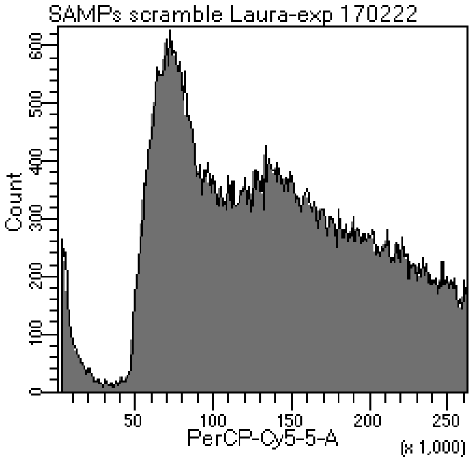

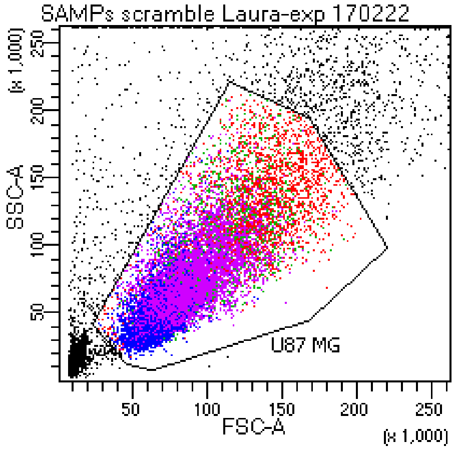

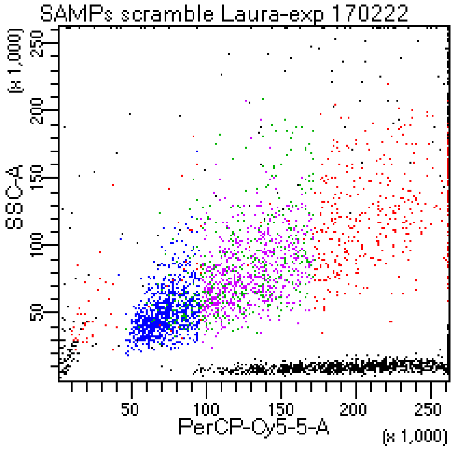

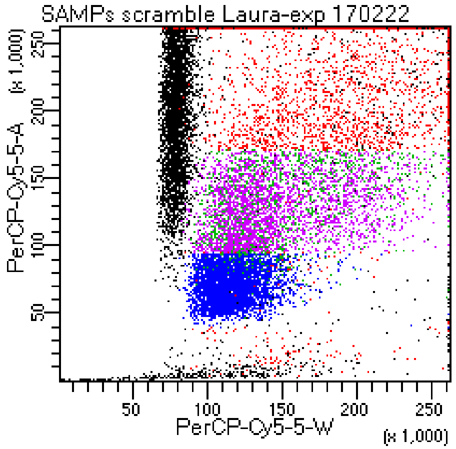

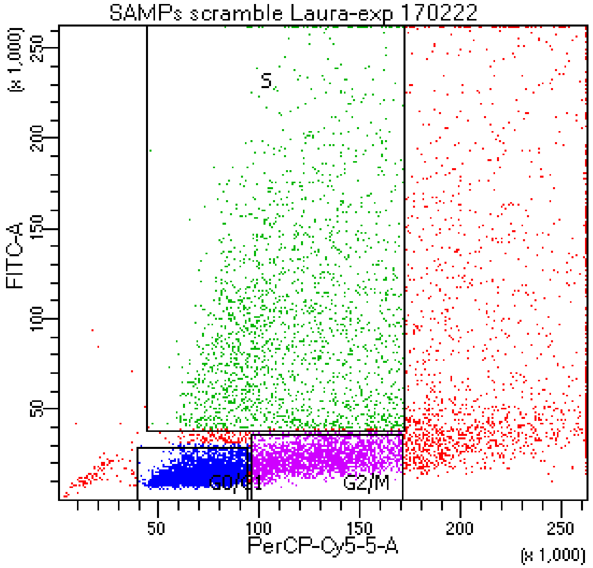

BD FACSDiva 8.0.1

Tube: exp 170222

Population	#Events	%Parent	%Total
All Events	87,482	####	100.0
U87 MG	49,751	56.9	56.9
S	9,929	20.0	11.3
G0/G1	17,577	35.3	20.1
G2/M	12,330	24.8	14.1

Experiment Name: U87 SAMPs -nanoparticelle...
Specimen Name: SAMPs scramble Laura
Tube Name: exp 170222
Record Date: Feb 17, 2022 4:40:41 PM
$OP: Administrator
GUID: d1a77da8-7312-46ba-9e1e...

Population	#Events	%Parent	FITC-A Mean	PerCP-C... Mean
U87 MG	49,751	56.9	47,076	118,560
S	9,929	20.0	103,120	116,373
G0/G1	17,577	35.3	13,953	69,511
G2/M	12,330	24.8	22,495	128,666
