# Supplementary figures and images for: Targeted inhibition of ubiquitin signaling reverses metabolic reprogramming and suppresses glioblastoma growth
Source: Commun Biol. 2022 Aug 2;5:780. doi: 10.1038/s42003-022-03639-8 (PMC9345969; doi:10.1038/s42003-022-03639-8)

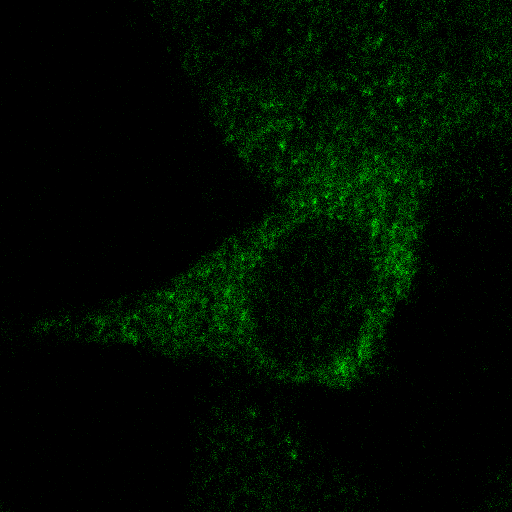

Supplement: Supplementary file 5 — Supplementary Data 2 [file 42003_2022_3639_MOESM5_ESM.zip › Supplementary Data 2/FIG 2D/GREEN.tif]

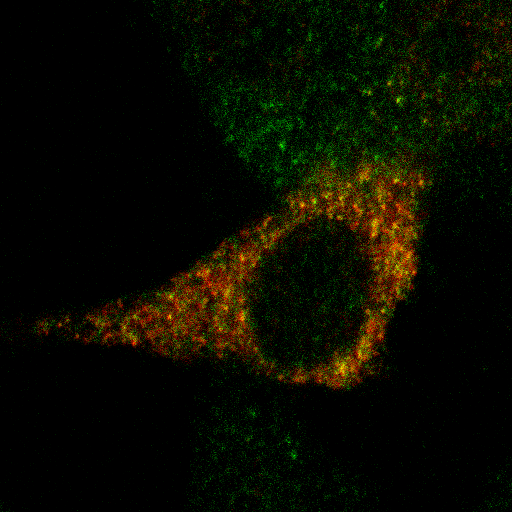

Supplement: Supplementary file 5 — Supplementary Data 2 [file 42003_2022_3639_MOESM5_ESM.zip › Supplementary Data 2/FIG 2D/MERGE.tif]

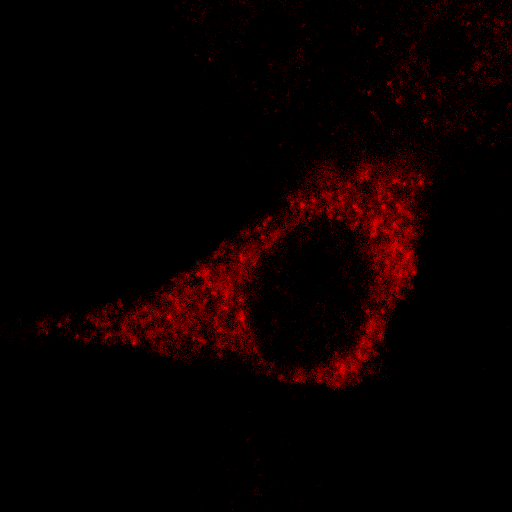

Supplement: Supplementary file 5 — Supplementary Data 2 [file 42003_2022_3639_MOESM5_ESM.zip › Supplementary Data 2/FIG 2D/RED.tif]
