# Supplementary material for: Targeted inhibition of ubiquitin signaling reverses metabolic reprogramming and suppresses glioblastoma growth
Source: Commun Biol. 2022 Aug 2;5:780. doi: 10.1038/s42003-022-03639-8 (PMC9345969; doi:10.1038/s42003-022-03639-8)
Supplement: Supplementary file 6 — Reporting Summary [file 42003_2022_3639_MOESM6_ESM.pdf]

nature portfolio

Corresponding author(s): Antonio Feliciello

Last updated by author(s): May 10, 2022

Reporting Summary

Nature Portfolio wishes to improve the reproducibility of the work that we publish. This form provides structure for consistency and transparency in reporting. For further information on Nature Portfolio policies, see our Editorial Policies and the Editorial Policy Checklist.

Statistics

For all statistical analyses, confirm that the following items are present in the figure legend, table legend, main text, or Methods section.

n/a	Confirmed	
☐	☒	The exact sample size (*n*) for each experimental group/condition, given as a discrete number and unit of measurement
☐	☒	A statement on whether measurements were taken from distinct samples or whether the same sample was measured repeatedly
☐	☒	The statistical test(s) used AND whether they are one- or two-sided<br>*Only common tests should be described solely by name; describe more complex techniques in the Methods section.*
☐	☒	A description of all covariates tested
☐	☒	A description of any assumptions or corrections, such as tests of normality and adjustment for multiple comparisons
☐	☒	A full description of the statistical parameters including central tendency (e.g. means) or other basic estimates (e.g. regression coefficient) AND variation (e.g. standard deviation) or associated estimates of uncertainty (e.g. confidence intervals)
☐	☒	For null hypothesis testing, the test statistic (e.g. *F*, *t*, *r*) with confidence intervals, effect sizes, degrees of freedom and *P* value noted<br>*Give P values as exact values whenever suitable.*
☒	☐	For Bayesian analysis, information on the choice of priors and Markov chain Monte Carlo settings
☒	☐	For hierarchical and complex designs, identification of the appropriate level for tests and full reporting of outcomes
☐	☒	Estimates of effect sizes (e.g. Cohen's *d*, Pearson's *r*), indicating how they were calculated

Our web collection on statistics for biologists contains articles on many of the points above.

Software and code

Policy information about availability of computer code

Data collection	CPTAC-GBM discovery cohort protein assay data, KEGG PATHWAY Database, IntAct Molecular Interaction Database (ftp://ftp.ebi.ac.uk/pub/databases/intact//2021-07-06/psimitab/intact.txt), STRING (used by stringApp (Cytoskape tool).<br>Quality check of sequenced reads was performed using the tool FASTQC v.0.11.9. Alignment on reference genome (GenCode release 36) was performed using STAR v. 2.7.5a with standard parameters. Quantification of expressed genes was performed using featureCounts v. 1.22.2 with standard parameters. Differential expression analysis was performed using DESeq2 v.1.34.0
Data analysis	ARACNE algorithm from the MINET R package, R version 3.6.3, Cytoskape version 3.7.2, stringApp (Cytoskape app)

For manuscripts utilizing custom algorithms or software that are central to the research but not yet described in published literature, software must be made available to editors and reviewers. We strongly encourage code deposition in a community repository (e.g. GitHub). See the Nature Portfolio guidelines for submitting code & software for further information.

Data

Policy information about availability of data

All manuscripts must include a data availability statement. This statement should provide the following information, where applicable:

- Accession codes, unique identifiers, or web links for publicly available datasets
- A description of any restrictions on data availability
- For clinical datasets or third party data, please ensure that the statement adheres to our policy

RNASeq data are available in ArrayExpress repository under the accession number: E-MTAB-11137

Field-specific reporting

Please select the one below that is the best fit for your research. If you are not sure, read the appropriate sections before making your selection.

☒ Life sciences ☐ Behavioural & social sciences ☐ Ecological, evolutionary & environmental sciences

For a reference copy of the document with all sections, see nature.com/documents/nr-reporting-summary-flat.pdf

Life sciences study design

All studies must disclose on these points even when the disclosure is negative.

Sample size	We made no sample-size calculations. For each set of experiment sample size was extimated to be adequate based on the magnitude and consistency of misurable differences among the groups analysed
Data exclusions	On principle, data were only excluded for failed experiement
Replication	Replicate experiments were successful
Randomization	Mice were randomly assigned to the experimental groups
Blinding	Investigators were not blinded during experiments in vivo and in vitro

Reporting for specific materials, systems and methods

We require information from authors about some types of materials, experimental systems and methods used in many studies. Here, indicate whether each material, system or method listed is relevant to your study. If you are not sure if a list item applies to your research, read the appropriate section before selecting a response.

Materials & experimental systems

n/a	Involved in the study	
☐	☒	Antibodies
☐	☒	Eukaryotic cell lines
☒	☐	Palaeontology and archaeology
☐	☒	Animals and other organisms
☐	☒	Human research participants
☒	☐	Clinical data
☒	☐	Dual use research of concern

Methods

n/a	Involved in the study	
☒	☐	ChIP-seq
☐	☒	Flow cytometry
☒	☐	MRI-based neuroimaging

Antibodies

Antibodies used	Materials and Methods Section provided with the manuscript contains information on all antibodies used in our study
Validation	We used only commercially available, validate antibody.

Eukaryotic cell lines

Policy information about cell lines

Cell line source(s)	Human embryonic kidney cells and U-87 cell line were purchased from ATTC.
Authentication	authentication of cell lines was carried out by ATTC
Mycoplasma contamination	Mycoplasm contamination was excluded using appropriate commercial kits (PCR and specific oligonucleotide primers) following the manufacturer's procedures
Commonly misidentified lines (See ICLAC register)	no cell line used are listed in the database of commonly misidentified cell lines

Animals and other organisms

Policy information about studies involving animals; ARRIVE guidelines recommended for reporting animal research

Laboratory animals	CD1 nude mice, female, 6 weeks old
Wild animals	no wild animals were used for experiments
Field-collected samples	the study do not involve samples collected from the fields
Ethics oversight	All animal experiments were performed in accordance to guidelines approved by Italian Ministry of Health (589/2017-PR; July, 21st 2017).

Note that full information on the approval of the study protocol must also be provided in the manuscript.

Human research participants

Policy information about studies involving human research participants

Population characteristics	Patients with diagnosis of Glioblastoma grade IV IDH1 wild type and IDH1R132 mutated, patients are all male age from 45 to 70
Recruitment	Neurosurgery IRCCS Neuromed
Ethics oversight	All male patients give their informed consent

Note that full information on the approval of the study protocol must also be provided in the manuscript.

Flow Cytometry

Plots

Confirm that:

- [x] The axis labels state the marker and fluorochrome used (e.g. CD4-FITC).
- [x] The axis scales are clearly visible. Include numbers along axes only for bottom left plot of group (a 'group' is an analysis of identical markers).
- [x] All plots are contour plots with outliers or pseudocolor plots.
- [x] A numerical value for number of cells or percentage (with statistics) is provided.

Methodology

Sample preparation	U87-MG cells were processed according to the BrdU Flow Kit protocol BD Pharmingen, following the manufacturer's procedures
Instrument	BD FACS "CANTO II" FLOW CYTOMETER
Software	BD FACS DIVA software are used for the experiment
Cell population abundance	Cells were not sorted
Gating strategy	Forward and side scatter gating; Pulse geometry gating; Subsetting gating

- [ ] Tick this box to confirm that a figure exemplifying the gating strategy is provided in the Supplementary Information.
